# The Microbe-Host Interface in Respiratory Tract Infections

**DOI:** 10.3201/eid1110.050831

**Published:** 2005-10

**Authors:** Matthew R. Moore

**Affiliations:** * Centers for Disease Control and Prevention, Atlanta, Georgia, USA

**Keywords:** respiratory tract infections, pathogenesis, immunity, book review

How thoroughly can 1 book address 2 complex aspects of the host-agent-environment triad, especially for a topic as broad as respiratory tract infections? Every author of an infectious disease topic assumes this task, at least implicitly. For example, the clinical aspects of adenovirus infection are hard to discuss without also highlighting the host factors that lead to greater susceptibility. The value of a book dedicated to the host-pathogen interaction depends on the book's ability to focus explicitly and narrowly on this relationship as the main topic.

Common to all 13 chapters of this first edition is the subject matter expertise of the authors. In addition to their thorough treatment of each subject, extensive referencing shows clearly the authors' command of current and past literature (in some instances, more space is devoted to references than to text). Beyond these common features, different chapters address particular facets of the host-agent relationship. Several chapters treat the host itself as the key subject, for example, the chapter on genetic background. Others place greater emphasis on the features of the microbes themselves, such as their pathogenicity and mechanisms for evading the host immune system. Still other chapters dissect and analyze every aspect of the complex relationship between host and agent, successfully making this interaction the central topic. The chapter on the pathogenesis of bacterial respiratory tract infections is a particularly strong example. Finally, some chapters look at the host-microbe interface over a period longer than the time of acute infection. For example, the chapter on atypical bacteria summarizes the evidence for a causal relationship between infection with *Mycoplasma pneumoniae* and the subsequent development of asthma.

If the authors' expertise is the primary strength of the book, the lack of organization and focus is its principal weakness. Most infectious disease textbooks adopt a pyramidal structure, beginning with foundational concepts, such as clinical syndromes, followed by specifics, such as the clinical presentation and treatment of individual pathogens. No such analogous structure is apparent in this book. Although the book begins with a discussion of genetics and the hygiene hypothesis, it quickly digresses into issues less relevant to the main point of the book, such as new diagnostic tests. A clear structure would also help ensure that all major topics are included. For example, many respiratory tract infections have a bimodal age distribution with the greatest incidence in the very young and the very old. However, this book largely omits any discussion of host-microbe interactions among the elderly. Similarly absent is a description of how pandemic influenza viruses emerge and evade the host immune system. Simply put, structure would unify what could otherwise be considered a series of well-done monographs.

Most readers who want to understand the host-agent interplay in respiratory infections might find that a general infectious disease text meets their needs. However, others who need more depth in selected topics should search the table of contents before adding this book to their library.

